# Using Bioactive Glasses in the Management of Burns

**DOI:** 10.3389/fbioe.2019.00062

**Published:** 2019-03-28

**Authors:** Saeid Kargozar, Masoud Mozafari, Sepideh Hamzehlou, Francesco Baino

**Affiliations:** ^1^Tissue Engineering Research Group (TERG), Department of Anatomy and Cell Biology, School of Medicine, Mashhad University of Medical Sciences, Mashhad, Iran; ^2^Bioengineering Research Group, Nanotechnology and Advanced Materials Department, Materials and Energy Research Center (MERC), Tehran, Iran; ^3^Cellular and Molecular Research Center, Iran University of Medical Sciences (IUMS), Tehran, Iran; ^4^Department of Tissue Engineering and Regenerative Medicine, Faculty of Advanced Technologies in Medicine, Iran University of Medical Sciences (IUMS), Tehran, Iran; ^5^Department of Medical Genetics, School of Medicine, Tehran University of Medical Sciences, Tehran, Iran; ^6^Medical Genetics Network (MeGeNe), Universal Scientific Education and Research Network (USERN), Tehran, Iran; ^7^Applied Science and Technology Department, Institute of Materials Physics and Engineering, Politecnico di Torino, Turin, Italy

**Keywords:** bioactive glasses, burns, wound healing, ion release, angiogenesis, antibacterial activity

## Abstract

The management of burn injuries is considered an unmet clinical need and, to date, no fully satisfactory solution exists to this problem. This mini-review aims to explore the potential of bioactive glasses (BGs) for burn care due to the therapeutic effects of their ionic dissolution products. BGs have been studied for more than 40 years and boast a long successful history in the substitution of damaged tissues, especially bone. Considering their exceptional versatility and attractive characteristics, these synthetic materials have also recently been proposed in the treatment of soft tissue-related disorders such as skin wounds. Specifically, improving fibroblast proliferation, inducing angiogenesis, and eliciting antibacterial activity (with the additional advantage of avoiding administration of antibiotics) are all considered as key added values carried by BGs in the treatment of burn injuries. However, some issues deserve careful consideration while proceeding with the research, including the selection of suitable BG compositions, appropriate forms of application (e.g., BG fibers, ointments or composite patches), as well as the procedures for reliable *in vivo* testing.

## Introduction

Burn injury is a frequent cause of morbidity and mortality over the globe. Based on a report published in 2016, about 486,000 patients in the US received medical care for burn injuries from 2008 to 2016; however, 3,275 of those passed away because of the severity of the injuries (Association, 2016). Based on the injury degree, a burn leads to several complications including infection, hypothermia, scarring, as well as bone and joint problems (Sevitt, 1979). Among the difficulties as mentioned above, bacterial infections are the leading cause of death (42–65%) after extensive burn injuries (Sharma et al., 2006; Bloemsma et al., 2008; Keen et al., 2010; Krishnan et al., 2013). It has been shown that Gram-positive and Gram-negative multidrug-resistant (MDR) bacteria contribute to the burn infections in the first and late post-injury days, respectively (Lachiewicz et al., 2017). Therefore, the treatment of burns is of great importance, and the slow healing process and hypertrophic scarring are, in fact, unresolved challenges in burn research and management (Wang et al., 2018).

After any burn, various therapeutic agents (e.g., antibiotics and silver-containing ointments) can be used in order to prevent infection from developing as well as to kill the actual microbial cells (see Table 1) (Dai et al., 2010). It has been noted that there is no need for the administration of prophylactic oral antibiotics in the case of minor burns since they may create multi-resistant bacteria (Branski et al., 2009; Barajas-Nava et al., 2013). In this situation, antibacterial dressings can keep the bacterial colonization of wounds to a minimum (Hyland et al., 2015). Furthermore, it has been reported that the use of therapeutic substances that induce angiogenesis can lead to accelerating the repair and regeneration of burn wounds (Galeano et al., 2006; Oryan et al., 2018).

**Table 1:** A short list of topical antimicrobial agents used for burn therapy.

**Agent class**	**Specific agent/Product**	**Application**	**References**
Topical antibiotics	Mafenide acetate	Clinical 2nd/3rd-degree burns	Haynes, 1971
	Bacitracin	Clinical 2nd/3rd-degree burns	Johnson et al., 1945
	Mupirocin	Clinical 2nd/3rd-degree burns	Palmieri and Greenhalgh, 2002
	Neosporin	Clinical 2nd/3rd-degree burns	Sinha et al., 1997
	Polymyxin B	Clinical 2nd/3rd-degree burns	Brown and Wood, 1972
	Nitrofurazone	Clinical 2nd/3 rd degree burns	Munster, 1984
	Nystatin	Clinical 2nd/3rd degree burns, fungal infections	Palmieri and Greenhalgh, 2002
Silver	Silver nitrate	Clinical 2nd/3rd-degree burns	Moyer et al., 1965
	Silver sulfadiazine	Clinical 2nd/3 rd degree burns	Fox, 1968
	Silver foams (Contreet, Allevyn)	Clinical 2nd/3 rd degree burns	Jørgensen et al., 2005
	Flammacerium	Clinical 2nd/3rd-degree burns	Monafo et al., 1976
	Acticoat 7	Clinical 2nd/3 rd degree burns	Fong and Wood, 2006
	Aquacel-Ag	Clinical 2nd/3rd degree burns	Barnea et al., 2010
	Silvercel	Clinical 2nd/3rd-degree burns	Meaume et al., 2005
	Silver amniotic membrane	Clinical 2nd/3 rd degree burns	Sawhney, 1989
Chitosan	Hydrogel	Clinical 2nd-degree burns	Ribeiro et al., 2009
	Film	2nd degree burns in rabbits	Sezer et al., 2008
	Bandage	Mouse burn infections (Psuedomonas, Proteus)	Dai et al., 2009
Antimicrobial peptide	Defensins	*In vitro*	Ganz, 2009
	Demegel	Pseudomonas infected rat burns	Chalekson et al., 2002
	Histone H1.2	Pseudomonas infected rat burns	Jacobsen et al., 2005
	Cecropin B	Pseudomonas infected mouse wounds	Ren et al., 2006
	rBPI	Clinical trial 2nd-degree burns	Steinstraesser et al., 2008
	Ceragenins	*In vitro*	Epand et al., 2008

*Reproduced with some modifications from (Dai et al., 2010)*.

**Figure 1:**
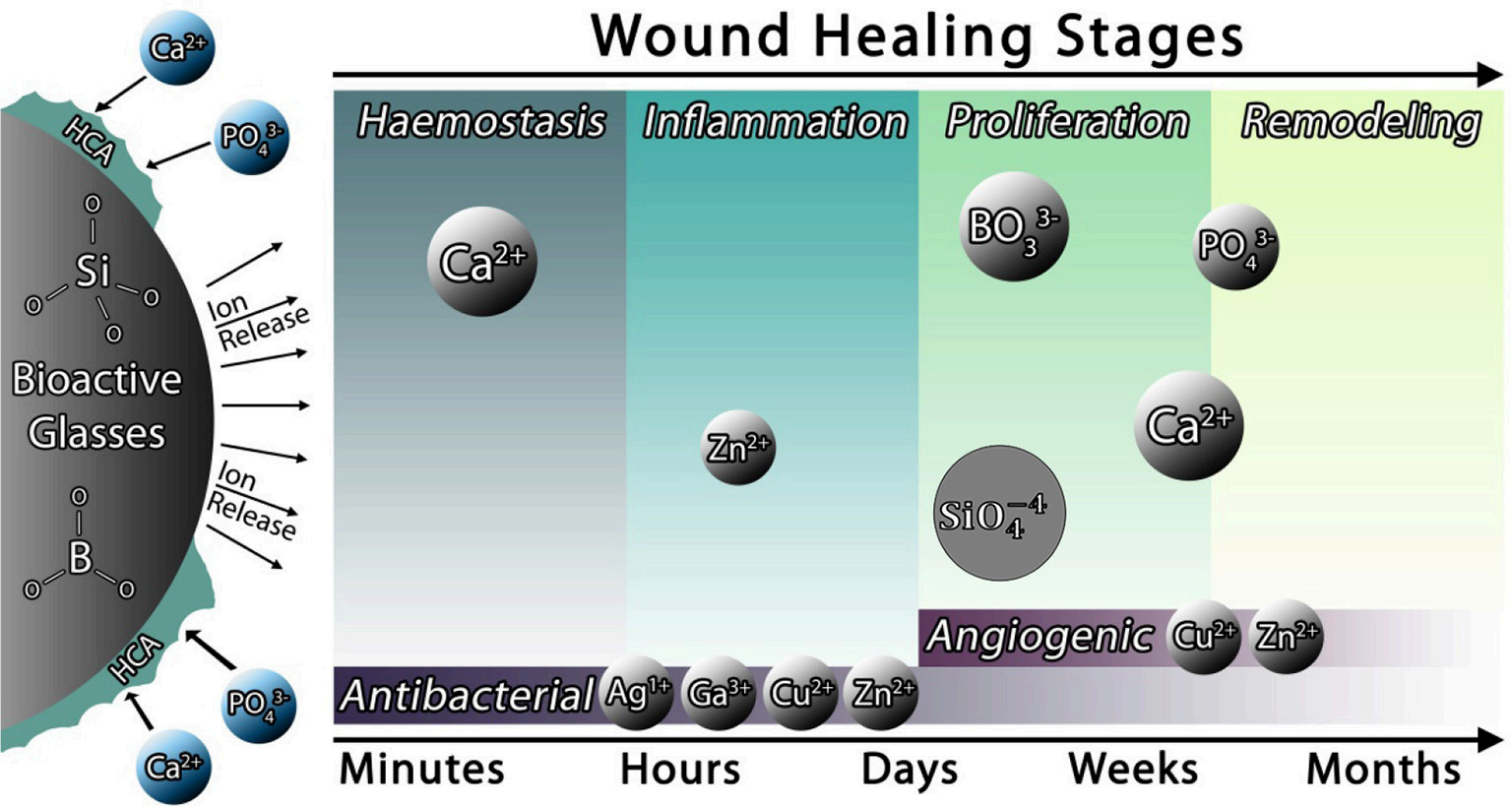
The release of some metal ions from BGs into the surrounding environment has a positive effect on wound healing. Reproduced with some modifications from Naseri et al. (2017).

According to the criteria mentioned above, a feasible treatment approach that may fulfill all these requirements together and be actually and safely applied in the clinical practice still remain to be developed. In this regard, bioactive glasses (BGs) have been recently identified as promising substances for the management of soft tissue-related disorders. These synthetic biomaterials have been using for the treatment of acute and chronic wounds (Naseri et al., 2017; Kargozar et al., 2019). Releasing various therapeutic ions from BG structure into the biological environment is the main reason for their positive effects on wound healing (see Figure 1) and, more specifically, the great potential of BGs (as a topical therapy) could be exploited to treat burn injuries as many topical antimicrobial agents used for burns are cytotoxic to soft tissue cells (e.g., keratinocytes and fibroblasts), thus resulting in an unwanted delay in wound healing process (Lineaweaver et al., 1985; Barsoumian et al., 2013). The positive effects of BGs on the cells (e.g., keratinocytes) involved in better and faster healing of the burn wounds have been well-understood (Mârza et al., 2019), bringing new hopes in this important area of science.

After providing a general picture of the suitability of BGs in contact with soft tissues – with emphasis on “general” wound healing applications (section Attractive Properties of BGs for Soft Tissue Repair) —, this mini-review highlights the potential of BGs in the context of burn treatment (section Evidence of BG Suitability for Treating Burns). Since there is a paucity of studies specifically dealing with BGs for the management of burns, an effort was done in section Evidence of BG Suitability for Treating Burns—whenever possible—to underline the specific relevance of other reports coming from the broader field of wound-healing applications.

## Attractive Properties of BGs for Soft Tissue Repair

After four decades from their invention, BGs have gained an important status in the biomedical field (Baino et al., 2018, 2019). They are routinely used for treating various diseases form bone injuries to cancer metastases (Johari et al., 2016; Kargozar et al., 2016, 2017a, 2018d; Miola et al., 2019). The composition of the first BG developed by Prof. Larry Hench (melt-derived 45S5 Bioglass®) is based on a four-oxide system, 45SiO_2_-24.5Na_2_O−24.5CaO−6P_2_O_5_ (wt %), which has a high amount of Na_2_O and CaO as well as a relatively high CaO/P_2_O_5_ ratio that makes the surface of the material very reactive in physiological environment. This reactivity provides the BGs with the capability of bonding to both hard and soft living tissues (Baino et al., 2016). Although the primary types of BGs (silicate glasses) were designed and used for hard tissue engineering, especially bone healing, the use of them for soft tissue healing has also been reported. As an illustration, Yu et al. clarified that pre-treatment of fibroblasts by a silicate BG could be an effective approach for the activation of skin tissue engineering constructs for improved wound healing (Yu et al., 2015). The *in vitro* and *in vivo* experiments showed that BGs stimulate fibroblasts to overexpress some important growth factors and proteins (e.g., VEGF, bFGF, EGF, collagen type I, and fibronectin) which leads to (Association, 2016) an improvement in their migration ability, (Sevitt, 1979) an increment in the blood vessel formation, and (Krishnan et al., 2013) the differentiation of fibroblasts into myofibroblasts in the wound site. All the mentioned events, regulated via pretreatment with BG, resulted in an acceleration of wound healing process. Moreover, the efficacy of BGs for wound healing was shown at the molecular level as reported by Li et al. (2016b). They showed that 45S5 Bioglass® ion extracts could prevent the death of human umbilical vein endothelial cells (HUVECs) following hypoxia, possibly through connexin hemichannel modulation. The positive effects of BGs on gap junction communication as well as the overexpression of connexin43 (Cx43) and other molecules involved in wound healing, e.g., VEGF and FGF, were also reported by the authors.

Recent advances have shown that new compositions of BGs (e.g., borate and phosphate glasses) can be applied for soft tissue healing (e.g., wound healing) as well (Naseri et al., 2017). Compared to silicate glasses, borate- and phosphate-based BGs exhibit some *in vitro* and *in vivo* behaviors (e.g., high dissolution rate) which are in favor of soft tissue applications and quick replacement with new tissue (Rahaman et al., 2011). In this regard, Hu et al. evaluated the efficiency of copper-doped borate BG/poly (lactic-*co*-glycolic acid) dressing loaded with vitamin E (0-3.0 wt.%) for full-thickness wound healing (Hu et al., 2018). The *in vitro* results clarified that the ions released from the dressings encouraged the migration, tubule formation, and VEGF secretion in HUVECs and fibroblasts. Moreover, the data obtained from *in vivo* experiments revealed a substantial improvement in the epithelialization of wound closure and a significant increase in vessel sprouting and collagen remodeling. The authors stated that the use of this composite biomaterial as a wound dressing could actually be a promise for accelerating the healing and reconstruction of full-thickness skin defects.

**Figure 2:**
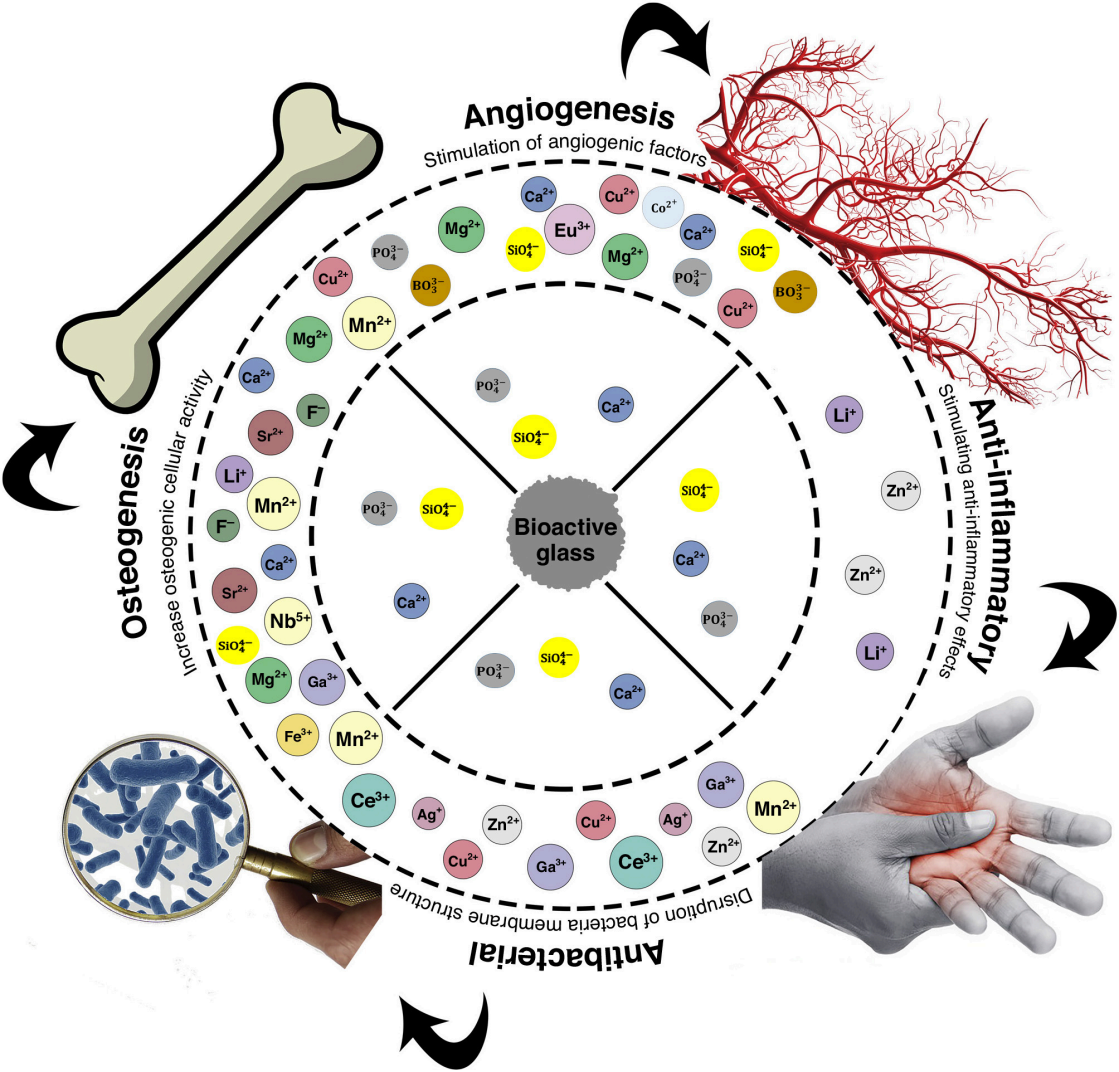
The biological effects of BGs are related to the release of therapeutic ions from their structure into the surrounding environment. Reproduced with some modifications from Kargozar et al. (2018a).

After being incorporated into the structure of BGs, some specific ions may be subsequently released and elicit beneficial biological effects such as improved cell proliferation (Xynos et al., 2000), inhibition of bacterial growth (Zhang et al., 2010), and stimulation of angiogenesis (Kargozar et al., 2017b) (see Table 2 and Figure 2).

**Table 2:** Therapeutic ions useful for soft tissue healing applications.

**Therapeutic ions**	**Application in wound healing**	**References**
Silver (Ag^+^)	Inhibition of bacterial growth and thereby prevention of infection	Lin et al., 2016a
Zinc (Zn^2+^)	Improving epidermal keratinocyte proliferation and migration	Deters et al., 2003; Lansdown et al., 2007
	Showing antioxidant effects	Rostan et al., 2002
	Inhibition of bacterial growth and thereby prevention of infection	Sirelkhatim et al., 2015
Copper (Cu^2+^)	Regulation of the activity of proteins involved in wound healing such as VEGF (enhancing angiogenesis) and maturation of collagen and elastin	Sen et al., 2002; Kornblatt et al., 2016
	Inhibition of bacterial growth and thereby prevention of infection	Abou Neel et al., 2005
Cerium (Ce^3+^)	Improving the proliferation and migration of fibroblasts, keratinocytes, and VECs	Chigurupati et al., 2013; Ramenzoni et al., 2017
	Showing antioxidant and anti-inflammatory effects	Davan et al., 2012; Kargozar et al., 2018c
	Inhibition of bacterial growth and thereby prevention of infection	Kaygusuz et al., 2017c
Gallium (Ga^3+^)	Anti-inflammatory effects	Whitacre et al., 1992; Orosz et al., 1996
	Inhibition of bacterial growth and thereby prevention of infection	Thompson et al., 2015
Calcium (Ca^2+^)	Improving hemostasis	Lansdown, 2002
	Modulation of keratinocyte proliferation and differentiation	Lansdown, 2002
	Improving fibroblast proliferation	Kawai et al., 2011
	Improving type I collagen synthesis and the increasing ratio of collagen I/III	Wang et al., 2015
Boron [(BO_3_)^3−^]	Acceleration of wound healing via activation of angiogenesis (overexpression of VEGF and TGF-β)	Balasubramanian et al., 2017
	Enhancing the proliferation, migration, and production of vital growth factors of dermal cells	Demirci et al., 2016

For example, incorporation of silver ions (Ag^+^) into the glass structure imparts antimicrobial properties over various Gram-positive and Gram-negative bacterial strains (e.g., *E. coli, P. aeruginosa*, and *S. aureus*), without eliciting any toxic effect on human cells if silver is added under a threshold dosage (Miola and Verné, 2016). The release kinetics of therapeutic ions from BGs into the surrounding environment is determined by some factors such as the ionic radius of doping element, type of glass network, pH of the host environment, and temperature (Kargozar et al., 2018a).

As one of the most attractive types of BGs, mesoporous BGs (MBGs) are also considered as promising candidates for repair and regeneration of wound injuries (Wang et al., 2016; Kargozar and Mozafari, 2018). Experimental evidence showed that these materials could be used as suitable carriers for the controlled delivery of various therapeutic ions, drugs, and chemicals accelerating the healing process. The usability of MBGs have been previously well documented for antibacterial strategies (Gargiulo et al., 2013; Wu et al., 2013; Kargozar et al., 2018d) and angiogenesis-requiring applications (Dashnyam et al., 2017; Zhou et al., 2017; Romero-Sánchez et al., 2018). Also, Pourshahrestani et al. reported that gallium-containing MBGs could improve hemostasis via stimulating blood coagulation, platelet adhesion and thrombus generations (Pourshahrestani et al., 2016).

In the context of soft tissue engineering, Wang et al. developed a biocomposite made of copper-containing MBGs and nanofibrillated cellulose (NFC) as a suitable dressing material for chronic wound healing application (Wang et al., 2016). They reported that this composite showed high bioactivity in simulated body fluid (SBF) and could act as a matrix for the sustained release of Cu^2+^ ions which inhibited the bacterial growth and improved angiogenesis in order to promote wound healing. Other MBG-containing polymer biocomposites like chitosan (CS)/MBG porous films have also been developed as promising wound dressing materials (Jia et al., 2011).

## Evidence of BG Suitability for Treating Burns

Generally, burns are categorized into four groups based on their depth and severity as 1st, 2nd, 3rd, and 4th degree. The extent of the injury is limited to only the epidermis in the first-degree burns, while it involves more layers of skin in the second-degree burns including the superficial (papillary) dermis and also the deep (reticular) dermis layer. The epidermis is lost, and damage to the subcutaneous tissue occurs in the case of third-degree burns; in addition to that, damage to the muscle, tendon, and ligament tissue are observed in the fourth-degree burns (Dai et al., 2010). It has been well documented that burns >10% of total body surface area (%TBSA) in children or 15% in adults are potentially life-threatening injuries as a result of the risk of hypovolemic shock (Malic et al., 2007).

A few criteria are typically counted for any medication used to treat or reduce the symptoms of burn injuries, i.e., attenuating inflammation, reducing infection, removing excessive exudates, and improving angiogenesis, thereby accelerating the healing process (Rowan et al., 2015). The promise of BGs in meeting these requirements is illustrated in Table 3. As previously mentioned, antimicrobial dressings are recommended for the management of minor burns (Hyland et al., 2015). However, it has been stated that there is no need for wound coverage if the skin is intact and not blistered. In this condition, the use of a simple moisturizer (e.g., silver sulfadiazine cream) has been recommended (Australian NZB Association, 2002). On the other hand, excessive or prolonged inflammation can result in impaired wound healing (Sommer et al., 2013). It is possible to prepare some compositions of BGs with the ability to reduce inflammation (Varmette et al., 2009) and bacterial infection (Coraça-Huber et al., 2014). As mentioned above, chronic inflammation is one of the main complications in patients with large burns which may impair wound healing (Ueno et al., 2005). The efficacy of 45S5 Bioglass® on human macrophages and monocytes has been previously studied *in vitro*, showing its potential in terms of attenuating inflammatory responses (Day and Boccaccini, 2005). Moreover, there is a hope to improve the anti-inflammatory effects of BGs through doping with some specific ions (e.g., Zn^2+^ and Li^+^).

**Table 3:** Potential capability of BGs to meet the criteria required for burn management.

**Properties**	**Notes**	**References**
Attenuation of inflammation	Promising results in the general context of wound healing	Day and Boccaccini, 2005; Varmette et al., 2009
Prevention/treatment of infection	Convincing results in the general context of soft tissue healing. One study has been reported in the context of burn management, showing the efficacy of BGs against multidrug-resistant bacterial strains typical of human infected burns.	Gholipourmalekabadi et al., 2016
Promotion of angiogenesis	Convincing results in the general context of bone tissue and soft tissue healing.	Lin et al., 2012, 2016b; Quinlan et al., 2015; Li et al., 2016a; Kargozar et al., 2018b
Removal of exudates	No evidence of that has been reported yet. This property is peculiar of MBGs and is believed to be possible due to the highly-porous texture of MBGs.	Gholipourmalekabadi et al., 2016
Re-epithelization	Promising results in the context of tissue engineering.	Wang et al., 2017

From the antibacterial point of view, it has been shown that BGs can decrease the risk of infections caused by both Gram-positive and Gram-negative strains (Liu et al., 2016a,b; Ottomeyer et al., 2016). This decrease can be achieved through two mechanisms (usually combined) regulated by the dissolution of BGs, including (i) the local increase of pH values in the injured site (due to the delivery of alkaline cations such as Ca^2+^ and Na^+^) and (ii) the release of antibacterial ions (e.g., silver, copper, zinc, cerium, and gallium) (Ratha et al., 2018; Bauer et al., 2019; Wajda et al., 2019). However, it has been documented that the effect of pH depends on the species of bacteria involved. For example, Wiegand et al. showed that *S. aureus* exhibits an increased sensitivity against silver nitrate with rising pH while *P. aeruginosa* exhibits a decreased sensitivity (Wiegand et al., 2015). In order to illustrate the potential suitability of BGs in the specific context of burn injuries, Gholipourmalekabadi et al. compared the antibacterial activity of silver- and fluoride-containing BGs vs. commonly-used antibiotics on multidrug-resistant bacterial strains (*K. pneumonia, S. aureus, E. coli*, and *P. aeruginosa*) isolated from patients with burns (Gholipourmalekabadi et al., 2016). Their results revealed that, although fluoride-doped BGs did not show any antibacterial activity against the tested bacteria, BGs doped with 1 and 2% of silver significantly inhibited the bacterial growth *in vitro* in all cases (inhibition zone up to 11 ± 1 mm). On the basis of this early experimental evidence, they concluded that silver-doped BGs could play an important role in the prevention of burn-associated infections, reduction of pain, and removal of excessive exudates. The capability of removing exudates is of great importance since they can fail the dressing to stick to the injured tissues and subsequently disrupt the wound healing process.

At present, other specific studies dealing with the efficacy of BGs against the typical bacteria of infected burns are not available in the literature; however, the potential suitability of BGs for the management of burns can be supported by other results from “general” wound healing applications.

**Figure 3:**
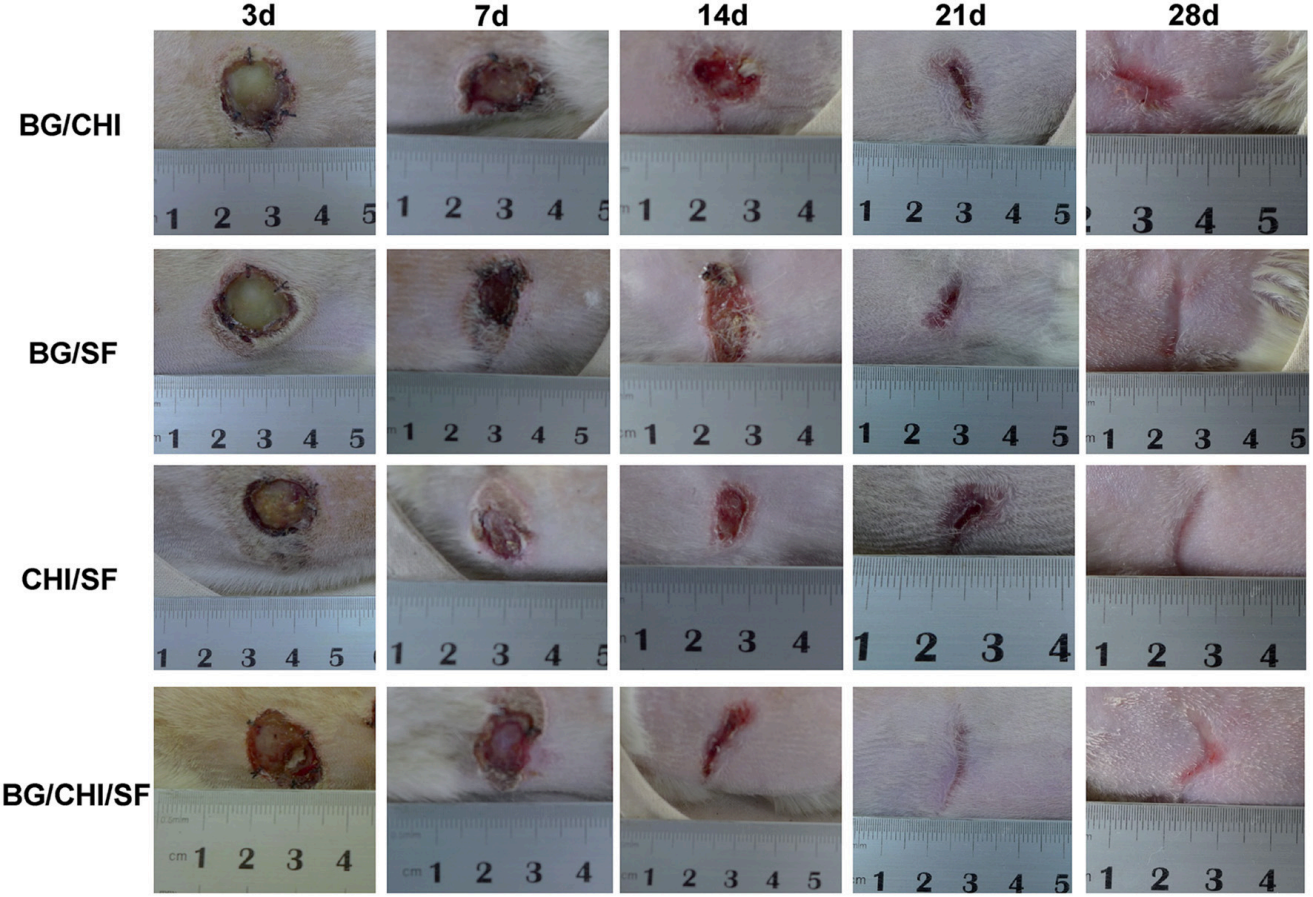
Direct observation of the burn wounds created in rats and treated with silk fibroin (SF)/chitosan (CHI), BG/SF, BG/CHI and BG/CHI/SF scaffolds after 3, 7, 14, 21, and 28 days. Reproduced with permission from Li et al. (2016a).

The formulation of BGs for use in wound injuries (and hence in burns) is of paramount importance. In this regard, Lin et al. prepared BG ointments by mixing 58S sol-gel BG micro-particles (SGBG-58S), 58S nano-BG (NBG-58S) and melt-derived 45S5 BG (45S5) powder with Vaseline (V) at 18 wt.% (Lin et al., 2012). They used this formulation for the treatment of full-thickness skin wounds in both normal and diabetic rats, whose injuries did not heal under conventional treatment. The obtained data revealed that all the compositions could accelerate the wound healing as a result of improving the proliferation of fibroblasts and growth of granulation tissue as well as the induction of angiogenesis. In addition to the susceptibility to infections, destruction of the vascular supply to the burned skin is one of the main barriers to the repair of injured tissue (Guo et al., 2011). Hence, improving angiogenesis regulated by BGs can be considered as a promising point regarding burn healing. The release of specific metal ions (e.g., copper and cobalt) from BGs into the surrounding environment can promote angiogenesis via hypoxia-mimicking pathways (Quinlan et al., 2015; Lin et al., 2016b; Balasubramanian et al., 2017; Kargozar et al., 2018b). Angiogenesis is vital to allow and/or accelerate the healing of burn wounds. On this matter, Li et al. developed BG/chitosan/silk fibroin composite scaffolds for the regeneration of deep burn wounds (Li et al., 2016a). The authors used these components with three specific and distinct aims: BG was included for inducing angiogenesis, chitosan for promoting the adsorption and enrichment of growth factors, and silk fibroin for providing a three-dimensional (3D) porous structure and mechanical support. Their results showed that adding BGs to the composites was the key to promote the formation and maturation of new blood vessels, which can significantly accelerate the wound healing process (see Figure 3).

Looking at the future and considering the peculiar properties that a biomaterial in contact with burns should exhibit, MBGs doped with therapeutic ions (see Table 2) could be regarded as ideal candidates being capable to act as multifunctional systems eliciting a local antibacterial effect and promoting angiogenesis, which are both crucial features required for the successful management of burns; furthermore, their highly-porous structure could be useful in adsorbing burn exudates.

As a further promising point, the sustained release of bioactive molecules that can be potentially effective in burn healing [e.g., epidermal growth factor (EGF)] via MBGs has been previously carried out; however, those studies were addressed to another application, i.e., the acceleration of bone regeneration (Wang et al., 2017).

## Implications and Conclusions

**Figure 4:**
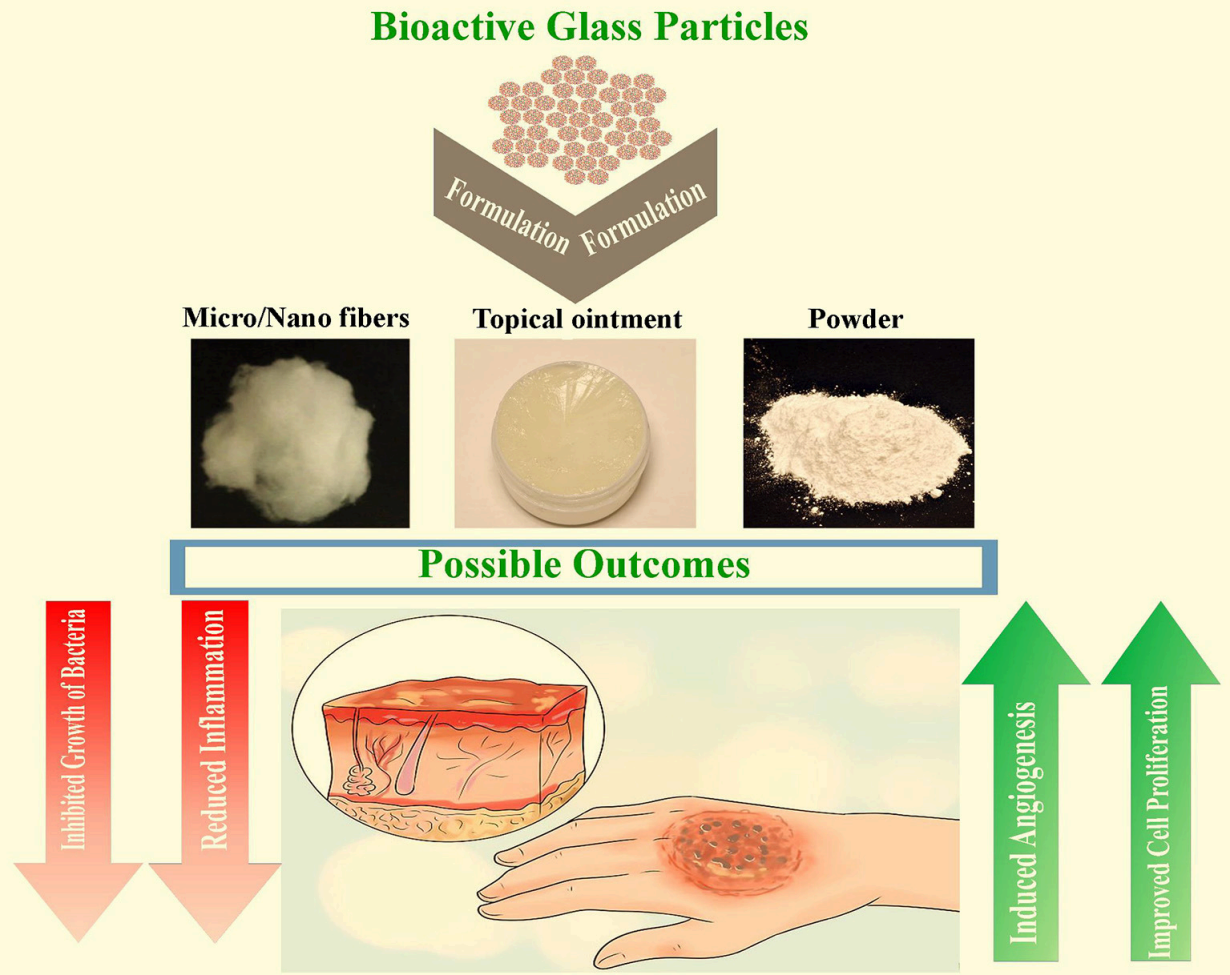
Schematic representation of possibilities of BGs for the treatment of burns. With some modifications from Homayoon (2015).

The impact of using BGs for the management of burns would be highly significant from multiple viewpoints including scientific, clinical, commercial, and socio-economical aspects. Specifically, MBGs show great promise due to their exceptional processing versatility and capability of acting as multifunctional platforms for the local release of therapeutic ions and biomolecules(Wu and Chang, 2014), which could perform a synergistic action (e.g., anti-inflammatory, antibacterial and angiogenic effects) addressed to promote the healing of burn injuries (see Figure 4). From a clinical perspective, the use of therapeutic inorganic agents (i.e., ionic dissolution products) released from BGs or MBGs to treat burn-related infections could ideally allow overcoming the problem of resistant bacterial strains, which has been associated to the abuse of antibiotics in the last decades and is one of the grand challenges of the 21st century (Ventola, 2015). In fact, bacteria cannot be resistant to the effects of some inorganic cations, such as Ag^+^, that typically cause the disruption of the membrane after linking to the membrane proteins (Silvestry-Rodriguez et al., 2007).

**Figure 5:**
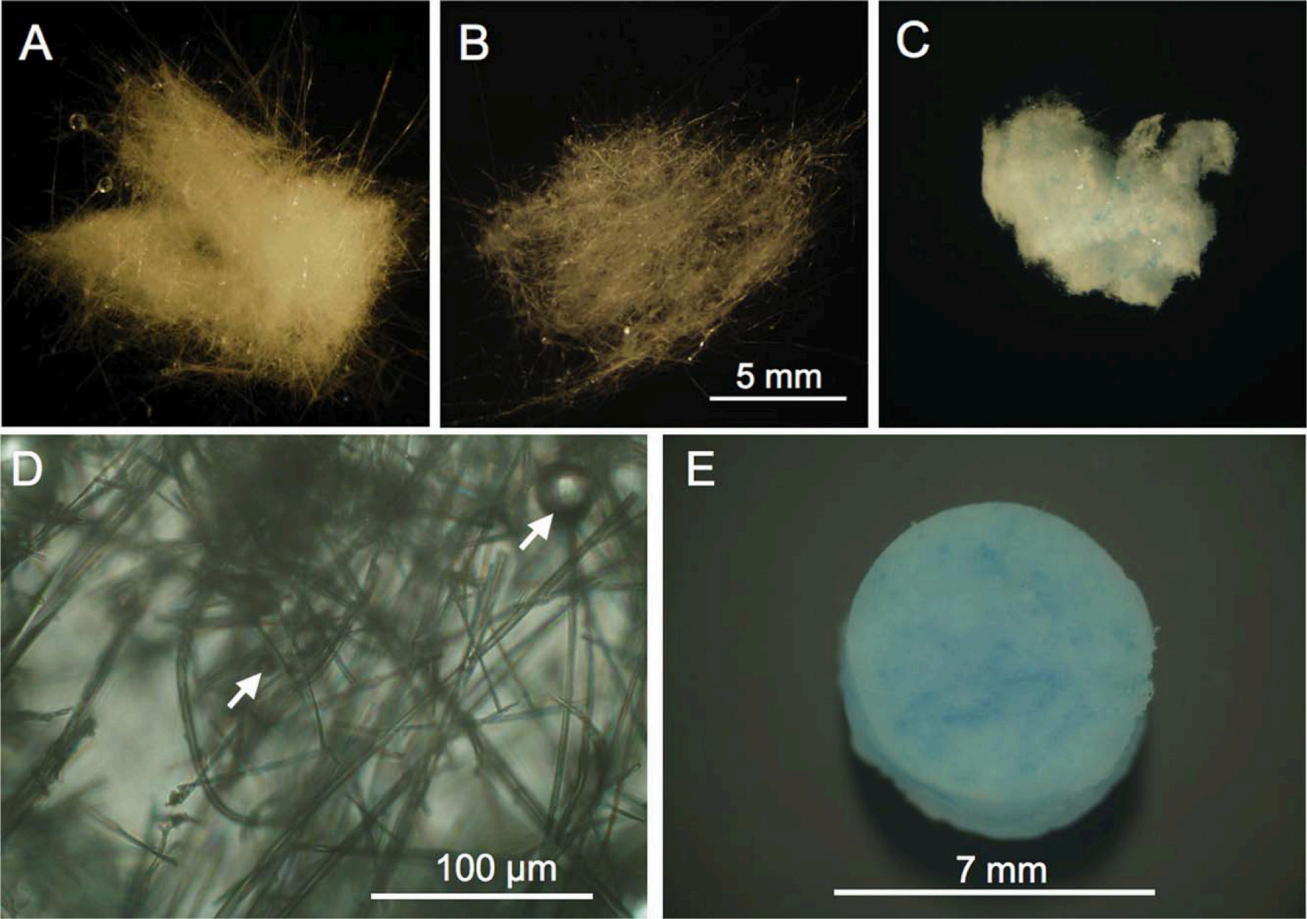
Cotton-like fibrous scaffolds produced and implanted subcutaneously in rats including **(A)** 45S5 glass, **(B)** 13–93B3 glass, and **(C)** Cu-doped 13–93B3 glass; **(D)** higher magnification of the 13–93B3 glass microfibers with glass beads of variable size; **(E)** the mat of copper-containing 13–93B3 glass microfibers for implantation in rats **(E)**. Images reproduced from Lin et al. (2014) with permission.

From a commercial viewpoint, BG-based medical products intended for the treatment of burn injuries would be unique and highly novel. At present, no BG-based commercial medication is available for this specific purpose. There is also a paucity of BG-based commercial products for “general” wound healing. Cotton-candy 13-93B3 borate BG (53B_2_O_3_-20CaO-12K_2_O-6Na_2_O-5MgO-4P_2_O_5_ wt.%, trade-named as “Dermafuse”) has been recently commercialized by Mo-Sci Corporation (USA) to accelerate wound healing in veterinary medicine and has also shown great promise for use in diabetic human patients suffering from chronic wounds (Wray, 2011). This material has a nanofibrous structure, so that blood platelets are trapped on the BG fibers that offer mechanical support and stability for tissue migration and the wound healing process. The fibers dissolve over time and form hydroxyapatite microspheres, to which blood vessels can attach. The antibacterial and proangiogenic properties of 13-93B3 BG formulation could justify the evaluation of this material for treating burn injuries, too. An evolution of this material was recently investigated by Lin et al. who successfully incorporates Cu^2+^ ions into 13-93B3 BGs microfibers and prepared wound dressings with an enhanced capability to stimulate angiogenesis, thereby significantly accelerating the healing of full-thickness skin defects in a rodent model (Lin et al., 2014) (Figure 5).

Appropriate *in vitro* and *in vivo* burn models should also be developed to test the suitability of BGs for this specific application; an overview of common testing methods is reported elsewhere (Qu and Nourbakhsh, 2017). It is stated that the method used to induce burns in experimental animals is, in fact, one of the most critical factors of clinical and ethical relevance; therefore, efforts should be made to reduce *in vivo* experiments to a minimum. In this regard, direct contact with a heated metal (usually created on the back of the animals) (Campelo et al., 2011), electricity (usually performed on large animals) (Zelt et al., 1988), and heated water (more widespread use) (Dahiya, 2009) are the commonly-used approaches to generate burn surfaces in experimental animal models (mouse, rat, pig, and monkey) (Abdullahi et al., 2014).

The selection of the most suitable form of application of BGs for burn care is another aspect deserving careful consideration. Surgeons typically claim an off-of-the-shelf and easy-to-use product that can fit the burn extension and homogeneously cover its surface. Three potential forms of application might be considered, i.e., fibrous BG constructs (such as the above-mentioned “Dermafuse”), BG-containing ointments that could be easily spread topically and pliable composites (for example BG micro- or nano-inclusions embedded in a soft polymeric sheet or gel) (Kargozar et al., 2018e). The feasibility of all these potential products has already been reported in the biomaterials literature or industry, although in other contexts than burn care: thus, no particular problems are expected to arise from a processing viewpoint due to the great “technological” versatility of BGs.

Last but not least, given the absence of biomolecules (drugs) incorporated in this new product for the treatment of burn injuries, the regulatory procedure required for clinical approval and use is expected not to be a tremendously draining and resource-consuming path, so that a large number of patients can benefit soon from these achievements.

## Data Availability

All datasets generated for this study are included in the manuscript and the supplementary files.

## Author Contributions

SK: writing the first draft; MM: revision; SH: preparation of Figures and Tables; FB: final revision.

### Conflict of Interest Statement

The authors declare that the research was conducted in the absence of any commercial or financial relationships that could be construed as a potential conflict of interest.
